# Assessment of Advanced Glycation End Products and Receptors and the Risk of Dementia

**DOI:** 10.1001/jamanetworkopen.2020.33012

**Published:** 2021-01-08

**Authors:** Jinluan Chen, Sanne S. Mooldijk, Silvan Licher, Komal Waqas, M. Kamran Ikram, André G. Uitterlinden, M. Carola Zillikens, M. Arfan Ikram

**Affiliations:** 1 Department of Internal Medicine, Erasmus University Medical Center, Rotterdam, the Netherlands; 2 Department of Epidemiology, Erasmus University Medical Center, Rotterdam, the Netherlands; 3 Department of Neurology, Erasmus University Medical Center, Rotterdam, the Netherlands

## Abstract

**Importance:**

Advanced glycation end products (AGEs) and their receptor (RAGE) are implicated in the pathophysiological processes of dementia and potentially underlie the association of diabetes with neurodegeneration. However, longitudinal studies examining this association are lacking.

**Objective:**

To determine whether markers of the AGE-RAGE system are associated with prevalent and incident dementia and with cognition.

**Design, Setting, and Participants:**

In this population-based cohort study including participants from the prospective Rotterdam Study, extracellular newly identified RAGE binding protein (EN-RAGE) and soluble RAGE (S-RAGE) were measured in plasma collected between 1997 and 1999 in a random selection of participants, and additionally in participants with prevalent dementia. Participants without dementia were followed up for dementia until 2016. Skin AGEs, measured as skin autofluorescence, and cognition were measured between 2013 and 2016 in participants without dementia. Data analysis was performed from June 2019 to December 2019.

**Exposures:**

EN-RAGE, S-RAGE, and skin autofluorescence.

**Main Outcomes and Measures:**

Prevalent and incident dementia and cognition, adjusted for potential confounders, including age, sex, diabetes, educational level, *APOE* ε4 carrier status, smoking, and estimated glomerular filtration rate.

**Results:**

Of 3889 included participants (mean [SD] age, 72.5 [8.9] years; 2187 [56.2%] women), 1021 participants had data on plasma markers (mean [SD] age 73.6 [7.8] years; 564 [55.2%] women), 73 participants had dementia at baseline, and during 10 711 person-years of follow-up, 161 participants developed incident dementia. Compared with low levels, high EN-RAGE level was associated with a higher prevalence of dementia (odds ratio [OR], 3.68 [95% CI, 1.50-8.03]; *P* = .003), while high S-RAGE level was associated with a lower prevalence of dementia (OR, 0.37 [95% CI, 0.17-0.78]; *P* = .01). These associations attenuated in a longitudinal setting, with hazard ratios of 0.65 (95% CI, 0.42-1.01) for high EN-RAGE (*P* = .05) and 1.22 (95% CI, 0.82-1.81) for high S-RAGE (*P* = .33). Among 2890 participants without dementia (mean [SD] age, 72.5 [9.4] years; 1640 [57%] women), higher skin autofluorescence was associated with lower global cognitive function (adjusted difference in *z* score per 1-SD higher skin autofluorescence, −0.07 [95% CI, −0.11 to −0.04]), especially among carriers of the *APOE* ε4 allele (adjusted difference in *z* score per 1-SD higher skin autofluorescence, −0.15 [95% CI, −0.22 to −0.07]).

**Conclusions and Relevance:**

These findings suggest that the AGE-RAGE system is associated with cognitive decline and dementia cross-sectionally but not longitudinally. This indicates either a short-term association or reverse causality. Findings of cross-sectional associations between higher skin autofluorescence and lower cognitive function and an association with *APOE* status also warrant replication and prospective studies.

## Key Points

**Question:**

Are advanced glycation end products (AGEs) and their receptor (RAGE) associated with cognition and dementia?

**Findings:**

In this cohort study among 3889 adults from the general Dutch population, markers of RAGE in plasma were associated with prevalent dementia in 1021 participants but were not associated with the incidence of dementia after a maximum of 18.7 years of follow-up. Skin AGEs were cross-sectionally associated with worse cognition in 2890 individuals.

**Meaning:**

These findings suggest that AGEs and RAGE were associated with the pathophysiological processes of cognitive decline and dementia, although their role in the long term should be further clarified.

## Teaser

This cohort study examines the association of advanced glycation end products and their receptor with dementia in the general Dutch population.

## Introduction

Diabetes is an established risk factor for dementia, but the exact mechanism remains unclear.^1^ Accelerated formation of advanced glycation end products (AGEs) has been proposed as a link.^2^ AGEs are a group of molecules generated nonenzymatically by attaching sugars to proteins, lipids, or nucleic acids and lead to modification and cross-linking of proteins.^3^ Furthermore, activation of the AGE receptor (RAGE) by AGEs or by other RAGE ligands, including amyloid-β, results in an inflammatory response and subsequently to upregulation of the receptor.^4^

Accumulation of AGEs takes place throughout life, especially in long-lived tissues. Excessive accumulation is observed in conditions of hyperglycemia and oxidative and inflammatory stress and is involved in diabetes, chronic kidney disease, and diseases associated with old age, including Alzheimer disease (AD).^5,6,7^ AGEs colocalize with AD-associated proteins in the brain, such as tau, neurofibrillary tangles, and amyloid-β.^7,8,9^ RAGE is also implicated in the pathophysiological processes of dementia and is thought to play a role in cerebral amyloid-β accumulation by facilitating its transport through the blood-brain barrier, as well as in neuronal degeneration and in the formation of fibrous tangles.^10^ Previous studies have found upregulation of RAGE expression in the brains of patients with AD.^11,12,13,14,15^ Furthermore, a recent study showed that markers of the AGE-RAGE system differ with *APOE* (OMIM 107741) ε4 carrier status, a genetic risk factor associated with dementia.^16^

AGEs can be measured in the skin using skin autofluorescence, a proxy associated with accumulation of AGEs in long-lived tissues, including the brain.^17,18^ Circulating molecules that are involved in the AGE-RAGE system include extracellular newly identified RAGE-binding protein (EN-RAGE), a RAGE ligand that has been associated with several chronic inflammatory diseases and coronary heart disease,^19,20^ and the soluble form of RAGE (S-RAGE), which acts as a decoy for RAGE ligands and may have an anti-inflammatory effect.^21^

To our knowledge, no studies have provided longitudinal information about the association of the AGE-RAGE system with dementia. To address this knowledge gap, we examined the association of plasma levels of EN-RAGE and S-RAGE with dementia cross-sectionally and longitudinally and the association of skin autofluorescence with cognition cross-sectionally.

## Methods

### Study Setting

This study was conducted within the Rotterdam Study, a prospective population-based cohort study that initiated in 1989 with 7983 participants aged 55 years and older (RS-I). As a second recruitment wave, 3011 new participants aged 55 years and older participated from 2000 onwards (RS-II). Another 3932 participants aged 45 years and older participated from 2006 onwards (RS-III). Follow-up examinations at the research center take place every 4 to 6 years. The objectives and further details of the study have been described elsewhere.^22,23^

This study follows the Strengthening the Reporting of Observational Studies in Epidemiology (STROBE) reporting guideline. The Rotterdam Study has been approved by the medical ethics committee of the Erasmus University Medical Center and by the review board of the Netherlands Ministry of Health, Welfare and Sports. All participants provided written informed consent.

### Study Population

EN-RAGE and S-RAGE were measured in plasma collected between 1997 and 1999 from a random subset of 964 participants of RS-I, and additionally of 57 participants with dementia (eFigure 1 in the Supplement). Skin autofluorescence was measured between 2013 and 2016 in 3009 participants from all 3 cohort waves. Of these, 2890 participants did not have dementia and underwent cognitive tests (eFigure 2 in the Supplement). Skin autofluorescence and cognitive test scores outside of the mean and 4 SDs range were considered outlying values and excluded from the analyses.

### Measurement of EN-RAGE and S-RAGE

Fasting blood samples were collected at the research center. Plasma was isolated and immediately put on ice and stored at −80 °C. Citrate plasma (200 μL) was sent in July 2008 to Rules-Based Medicine (Myriad RBM), where EN-RAGE and S-RAGE were assessed using multiplex immunoassay on a custom-designed human multianalyte profile. The intra-assay variability was less than 4%, and the interassay variability was less than 13%.

### Measurement of Skin Autofluorescence

During the visit to the research center, skin autofluorescence was measured at the inner side of the dominant forearm using the AGE Reader device (DiagnOptics) based on the fluorescent property of AGEs.^24^ The device has been validated against AGEs measured in skin biopsies from the same site where skin autofluorescence was measured.^17^ Participants were asked not to use skin creams before the measurement. The mean of 3 consecutive measurements was used for analyses.

### Assessment of Dementia

The Mini-Mental State Examination and the Geriatric Mental Schedule organic level were used to screen for dementia at baseline and subsequent center visits.^25^ Cutoffs were less than 26 for the Mini-Mental State Examination and greater than 0 for the Geriatric Mental Schedule. Participants with a positive screening outcome underwent further testing using the Cambridge Examination for Mental Disorders of the Elderly. Additionally, the electronic medical records from general practitioners and the regional institute for outpatient mental health care were used for the dementia diagnosis. The final diagnosis was established by a consensus panel led by a consultant neurologist, according to standard criteria for dementia (using *Diagnostic and Statistical Manual of Mental Disorders* [Third Edition Revised]^26^) and AD (using National Institute of Neurological and Communicative Disorders and Stroke-Alzheimer Disease and Related Disorders Association^27^).

### Cognitive Test Battery and General Cognition

During center visits, participants underwent a battery of cognitive function examinations assessing various cognitive domains, including the Letter Digit Substitution Task, the Stroop test, and the Word Fluency Test for executive function; the 15-Word Learning Test for memory; and the Purdue Pegboard test for fine motor skill.^28^ For tests consisting of subtasks, the interference task of the Stroop test, the delayed recall task of the Word Learning Test, and the sum score of all 3 tasks of the Purdue Pegboard were used for analyses.

### Assessment of Covariates

Information on alcohol use, smoking status, and educational level was obtained during structured home interviews.^22,29^ Body mass index was calculated as weight in kilograms divided by height in meters squared. Blood pressure was measured in the sitting position on the right arm using a random-zero sphygmomanometer. Serum concentrations of total cholesterol, high-density lipoprotein cholesterol, triglycerides, glucose, and creatinine were measured in fasting blood samples. The estimated glomerular filtration rate (eGFR) was calculated using the Chronic Kidney Disease Epidemiology Collaboration equation.^22,30^ *APOE* was genotyped by polymerase chain reaction in RS-I and by biallelic TaqMan assay in RS-II and RS-III.^31,32^ Participants were categorized as carries of 0, 1, or 2 ε4 alleles according to the *APOE* genotype. Diabetes was defined as fasting blood glucose greater than 126.13 mg/dL (to convert to millimoles per liter, multiply by 0.0555) or use of antidiabetic medications or self-reported as having diabetes.^33^ Chronic kidney disease was defined as eGFR less than 60 mL/min/1.73 m^2^. Depressive symptoms were considered as a score of 16 or higher on the validated Center for Epidemiology Depression Scale.^34^

### Statistical Analysis

EN-RAGE and S-RAGE were analyzed continuously, per SD increase of log transformed values because of skewed distributions, and categorized into tertiles, with the lowest tertile as the reference. Prior to analysis, outliers were excluded (defined as outside of the mean and 4 SDs range). To assess the association of EN-RAGE and S-RAGE with dementia, we used logistic regression for the cross-sectional analyses and Cox proportional hazard models for the longitudinal analyses. Follow-up started when blood was drawn and ended at the date of dementia diagnosis, date of death, or end of the study period (January 1, 2016), whichever came first. Follow-up until January 1, 2016 was near complete (10 711 of 11 079 [97%] potential person-years). A timeline for data collection is shown in eFigure 3 in the Supplement. Furthermore, we examined how these associations changed over follow-up time by performing analyses in incremental periods of follow-up.^35^ Briefly, participants who did not develop dementia were artificially censored at 4, 8, and 12 years; thus, the follow-up periods vary as 0 to 4 years, 0 to 8 years, 0 to 12 years, and 0 until the end of follow-up. In addition, we visualized overall survival during follow-up by EN-RAGE and S-RAGE tertile in Kaplan-Meier survival curves.

Skin autofluorescence values were analyzed per SD difference (the equivalent of 0.49 AU), after excluding outliers. Scores of the Stroop test were inverted; thus, higher scores for all cognition tests correspond to better performance. Cognitive test scores were standardized and analyzed as *z* scores. A parameter for general cognitive function, the G-factor, was calculated for participants with all test scores available by extracting the first principal component from the test scores of Letter Digit Substitution Task, Stroop (interference task), Word Fluency Test, Word Learning Test (delayed recall task), and the Purdue Pegboard sum score.^28^ This calculation was based on data from 3955 Rotterdam Study participants who had completed the full battery of tests. The cross-sectional associations of skin autofluorescence with the G-factor and with the individual tests were investigated in linear regression models. Although it was not the focus of this study, we additionally assessed the association of skin autofluorescence with prevalent dementia.

All analyses were adjusted for age, sex, and diabetes (model 1).^1,2,36^ Model 2 additionally adjusted for other potential confounders, selected based on literature, namely educational level, *APOE* ε4 carrier status, smoking behavior, and eGFR. To explore the association of covariates that are potential confounders but which may be intermediates as well, we additionally adjusted for blood pressure, high-density lipoprotein cholesterol level, total cholesterol level, triglyceride levels, lipid-lowering medication use, and depressive symptoms in model 3.^25,37,38,39^

#### Stratification and Sensitivity Analyses

To evaluate potential association modification by sex and *APOE* ε4 carrier status, their interaction with the AGE-RAGE markers was tested, and stratified analyses were performed. We also repeated the analyses stratified by subcohort and after excluding participants with diabetes and with chronic kidney disease to investigate whether these subgroups drove the associations. To evaluate whether age was sufficiently adjusted for, we tested whether additionally adjusting for age squared changed the results. Finally, we repeated the analyses restricting to dementia of the AD subtype.

Of all participants, 578 (15%) had at least 1 missing value (eTable 1 in the Supplement). Missing data on covariates were imputed using 5-fold multiple imputation (ie, multivariate imputation by chained equations package in R statistical software version 3.6.1 [R Project for Statistical Computing]).^40^ Survival analyses were conducted using the survival package in R. All other analyses were conducted using R Studio version 1.0.153. Statistical testing was performed 2-sided with *P* < .05 considered significant. Data were analyzed June 2019 to December 2019.

## Results

### EN-RAGE, S-RAGE, and Dementia

Among 7983 participants of the Rotterdam Study, 4214 participants (mean [SD] age, 72.8 [7.4] years; 2465 [58%] women) visited the research between 1997 and 1999. Of them, 1021 (mean [SD] age, 73.6 [7.8] years; 564 [55%]) had data on EN-RAGE and S-RAGE levels and were included in this study. For the cross-sectional analyses, 964 participants from the random subset with plasma data (mean [SD] age 73.0 [7.5] years; 529 [54.9%] women; 16 participants [1.7%] with dementia at baseline) and an additional 57 patients with dementia at baseline from the extended subset were eligible. The mean (SD) age at dementia diagnosis was 79.9 (7.9) years. After excluding participants with prevalent dementia and those without follow-up time, 945 participants were included in the longitudinal analyses (eFigure 1 in the Supplement).

Baseline characteristics of all patients with dementia at baseline and of the study population at risk for dementia are shown in Table 1. The value ranges for the EN-RAGE tertiles were less than 1.38 ng/mL in the lowest tertile, 1.38 ng/mL to 8.88 ng/mL in the middle tertile, and greater than 8.89 ng/mL in the highest tertile, and the teriles for S-RAGE were less than 2.15 ng/mL in the lowest tertile, 2.15 ng/mL to 3.22 ng/mL for the middle tertile, and 3.22 ng/mL or greater for the highest tertile. Compared with low levels, high EN-RAGE levels were associated with higher dementia prevalence (model 2 adjusted odds ratio [OR], 3.68 [95% CI, 1.50-8.03]; *P* = .003), and high S-RAGE levels were associated with lower dementia prevalence (model 2 adjusted OR, 0.37 [95% CI, 0.17-0.78]; *P* = .01) (Table 2). During a total follow-up of 10 711 person-years (median [interquartile range], 12.4 [6.2-16.3] years), 161 participants developed dementia (mean [SD] age at diagnosis, 84.4 [6.0] years), of whom 130 participants (81%) developed AD. We found no significant difference in risk of incident dementia among participants with a high EN-RAGE level compared with those with a low level (model 2 adjusted hazard ratio [HR], 0.65 [95% CI, 0.42-1.01]; *P* = .05), nor for participants with high S-RAGE levels compared with those with low levels (model 2 adjusted HR, 1.22 [95% CI, 0.82-1.81]; *P* = .33) (Table 2). There was no statistically significant interaction with sex or *APOE* ε4 carrier status (eTable 2 in the Supplement).

**Table 1.** Characteristics of the Study Populations for the Analyses With Cognition Data, Prevalent Dementia, and Incident Dementia

Characteristic	Participants, No. (%)^a^		
	With RAGE data		Cognition analysis subset (n = 2890)
	History of dementia (n = 73)	At risk for dementia (n = 945)	
Age, mean (SD), y	82.5 (8.2)	72.9 (7.4)	72.5 (9.4)
Women	45 (62)	518 (55)	1640 (57)
White race	68 (96)	909 (98)	2675 (96)
Educational level			
Primary	37 (51)	150 (16)	193 (7)
Lower	18 (25)	410 (44)	1120 (39)
Intermediate	14 (19)	292 (31)	871 (31)
Higher	3 (4)	85 (9)	662 (23)
*APOE* ε4 carrier status			
No *APOE* ε4 allele	27 (40)	651 (72)	1977 (73)
1 allele	30 (44)	243 (27)	672 (25)
2 alleles	11 (16)	14 (2)	63 (2)
Alcohol use	37 (61)	766 (82)	2471 (86)
Smoking			
Never	5 (8)	134 (14)	916 (32)
Former	26 (43)	474 (51)	1565 (55)
Current	30 (49)	328 (35)	373 (13)
BMI, mean (SD)	25.3 (3.3)	26.8 (3.9)	27.5 (4.3)
Blood pressure, mean (SD), mm Hg			
Systolic	138.8 (21.9)	144.0 (21.5)	144.6 (21.5)
Diastolic	71.49 (11.0)	75.05 (11.0)	83.71 (10.8)
Total cholesterol, mg/dL	255 (41)	258 (46)	211 (42)
High-density lipoprotein cholesterol, mg/dL	49 (15)	53 (15)	58 (17)
Triglycerides, median (IQR), mg/dL	88 (65-123)	85 (54-126)	113 (86-153)
Using lipid-lowering medication	3 (4.3)	118 (13.9)	877 (31)
Estimated glomerular filtration rate, median (IQR), mL/min/1.73 m^2^	65 (52-80)	73 (64-83)	75 (66-84)
Chronic kidney disease	25 (36)	158 (17)	413 (15)
Diabetes	14 (20)	105 (12)	393 (14)
History of cardiovascular disease and heart failure	10 (14)	59 (6)	266 (9.2)
Depressive symptoms	9 (21)	50 (6)	640 (22)
Plasma EN-RAGE, median (IQR), ng/mL	17.3 (11.8-22.2)	10.9 (7.8-14.8)	NA
Plasma S-RAGE, median (IQR), ng/mL	2.2 (1.6-3.1)	2.7 (2.0-3.7)	NA
Skin autofluorescence, AU	NA	NA	2.40 (0.49)

Abbreviations: EN-RAGE, extracellular newly identified RAGE binding protein; IQR, interquartile range; RAGE, advanced glycation end products receptor; S-RAGE, soluble RAGE.

SI conversion factors: To convert total and high-density lipoprotein cholesterol to millimoles per liter, multiply by 0.0259; To convert triglycerides to millimoles per liter, multiply by 0.0113.

^a^ Values are shown for nonimputed data.

**Table 2.** EN-RAGE and S-RAGE in Association With Prevalent and Incident Dementia

Measure	Participants with Dementia, No./Total No.	Model 1^a^	Model 2^b^	Model 3^c^
**Plasma EN-RAGE level**				
Dementia prevalence, OR (95% CI)				
Low	9/324	1 [Reference]	1 [Reference]	1 [Reference]
Medium	16/337	1.76 (0.74-4.19)	1.42 (0.55-3.64)	1.46 (0.55-3.85)
High	47/356	4.01 (1.86-8.68)	3.68 (1.50-8.03)	3.38 (1.42-8.04)
Per 1-SD increase	72/1017	1.80 (1.39-2.34)	1.74 (1.28-2.35)	1.67 (1.22-2.27)
Dementia incidence, HR (95% CI)				
Low	61/314	1 [Reference]	1 [Reference]	1 [Reference]
Medium	64/320	1.13 (0.79-1.61)	1.08 (0.76-1.55)	1.08 (0.75-1.56)
High	35/308	0.68 (0.45-1.05)	0.65 (0.42-1.01)	0.65 (0.42-1.00)
Per 1-SD increase	160/942	0.89 (0.76-1.05)	0.88 (0.74-1.04)	0.87 (0.74-1.03)
**Plasma S-RAGE level**				
Dementia prevalence, OR (95% CI)				
Low	35/346	1 [Reference]	1 [Reference]	1 [Reference]
Medium	20/341	0.54 (0.29-1.00)	0.56 (0.28-1.13)	0.61 (0.29-1.29)
High	17/333	0.50 (0.26-0.95)	0.37 (0.17-0.78)	0.35 (0.16-0.76)
Per 1-SD increase	72/1020	0.71 (0.55-0.92)	0.60 (0.45-0.81)	0.61 (0.45-0.84)
Dementia incidence, HR (95% CI)				
Low	51/309	1 [Reference]	1 [Reference]	1 [Reference]
Medium	54/321	0.93 (0.63-1.37)	1.04 (0.70-1.55)	1.00 (0.67-1.50)
High	56/315	1.09 (0.74-1.60)	1.22 (0.82-1.81)	1.15 (0.77-1.71)
Per 1-SD increase	161/945	0.94 (0.79-1.10)	0.94 (0.79-1.10)	0.95 (0.80-1.13)

Abbreviations: EN-RAGE, extracellular newly identified RAGE binding protein; HR, hazard ratio; OR, odds ratio; RAGE, advanced glycation end products receptor; S-RAGE, soluble RAGE.

^a^ Adjusted for age, sex, and diabetes.

^b^ Adjusted for potential confounders (ie, age, sex, diabetes, education, *APOE* ε4 carrier status, smoking status, and estimated glomerular filtration rate).

^c^ Adjusted for potential mediators (ie, systolic and diastolic blood pressure, cholesterol, high-density lipoprotein cholesterol, triglycerides, lipid-lowering medication use, and depressive symptoms) in addition to model 2.

**Figure 1.**
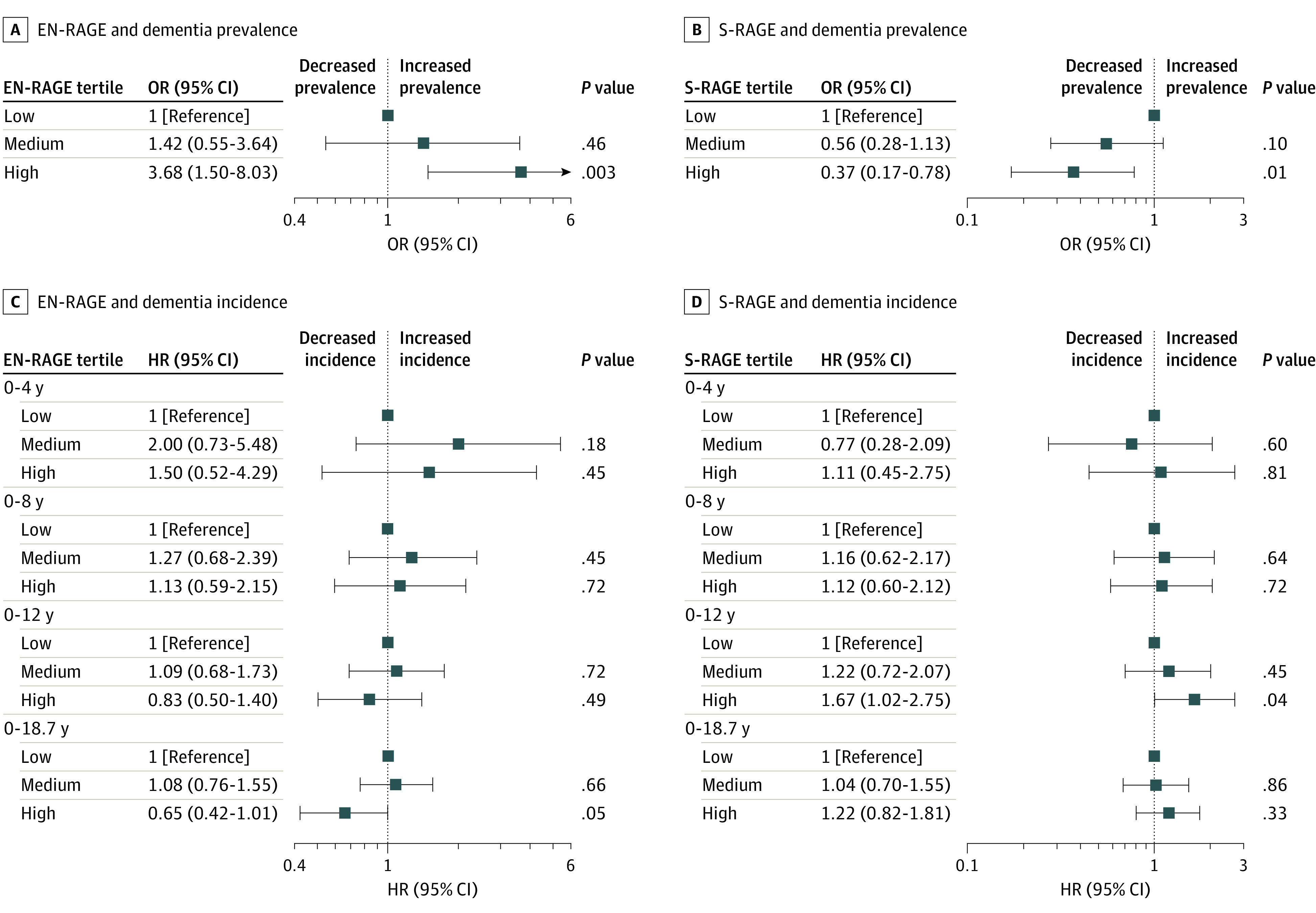
Association of EN-RAGE and S-RAGE With Prevalent and Incident Dementia by Time Intervals Associations of plasma levels with dementia incidence per cumulatively increasing duration of follow-up were obtained by censoring all participants still at risk at 4, 8, and 12 years after baseline, and after a total follow-up of 18.7 years. All estimates were adjusted for age, sex, diabetes, education, *APOE* ε4 carrier status, smoking, and estimated glomerular filtration rate. RAGE indicates Glycation End Products Receptor; EN-RAGE, extracellular newly identified RAGE binding protein; S-RAGE, soluble RAGE; HR, hazard ratio; and OR, odds ratio.

When analyzing in cumulative follow-up intervals from baseline, the risk of dementia for the high EN-RAGE group was higher in the first years compared with the low EN-RAGE group. With longer follow-up duration, this association of high EN-RAGE level diluted and even changed direction (Figure 1A and C). A lower risk of dementia with higher S-RAGE level was found for a short follow-up. However, with longer follow-up duration, this association also changed in direction (Figure 1B and D; eTable 3 in the Supplement). The survival curves showed that participants with high levels of EN-RAGE had lower survival than participants with medium and low levels. No differences in survival were found for the S-RAGE groups (eFigure 4 and eFigure 5 in the Supplement).

### Skin Autofluorescence and Cognitive Function

**Figure 2.**
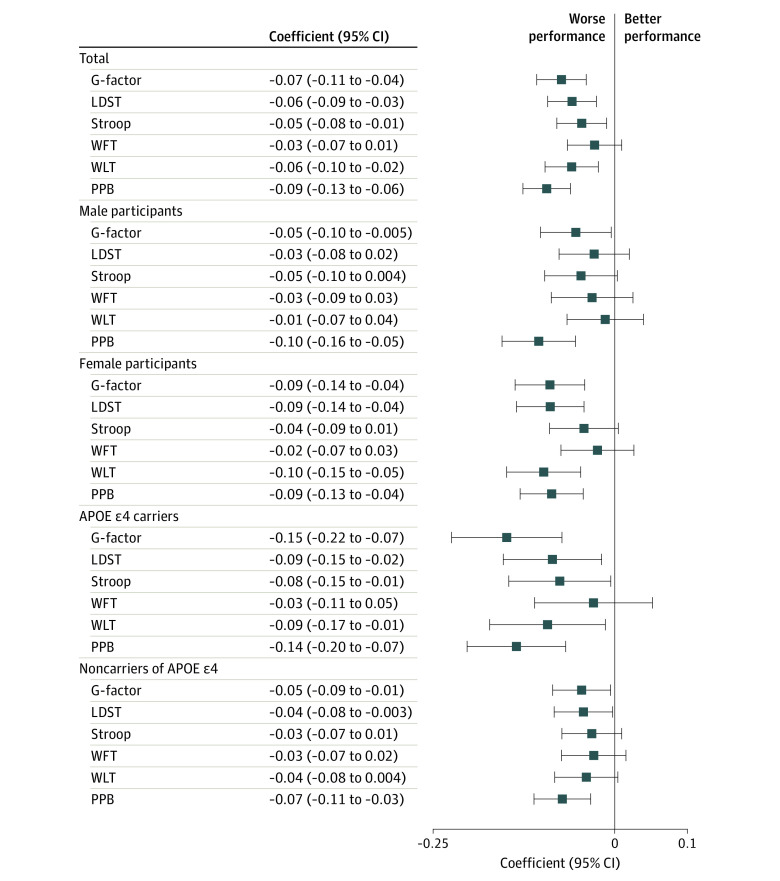
Associations of Skin Autofluorescence With Cognitive Function LDST indicates letter-digit substitution task; WFT, verbal fluency test; WLT, word learning test (delayed recall); and PPB, sum score Purdue pegboard tests, including tests on left hand, right hand, and both hands. The original score of the Stroop test (interference task) was inversely transformed. After transformation, a higher score corresponds to better performance. Coefficients are the difference in G-factor and *z*-scores of test parameters in association with 1-SD difference in skin autofluorescence, obtained from the linear regression adjusting for age, sex, diabetes, education, *APOE* ε4 carrier status, smoking status, and estimated glomerular filtration rate. For subgroup analysis by *APOE* ε4 carrier status, *APOE* ε4 carrier status was not used in the model for adjustment.

A total of 2890 participants who had undergone at least 1 of the cognitive tests and had a skin autofluorescence measurement available were eligible for skin autofluorescence analysis (mean [SD] age, 72.5 [9.4] years; 1640 [57%] women; mean [SD] skin autofluorescence, 2.40 [0.49] AU) (Table 1). Characteristics by skin autofluorescence tertiles are presented in eTable 4 in the Supplement, and details of tests are provided in eTable 5 in the Supplement. The G-factor explained 54.0% of the variance in cognitive test scores. Higher skin autofluorescence was associated with lower general cognitive function (model 2 adjusted mean difference in G factor per 1-SD higher skin autofluorescence, −0.07 [95% CI, −0.11 to −0.04]), and with the worse performance in individual tests. The associations were stronger among individuals who were *APOE* ε4 carriers than among noncarriers (adjusted difference in G factor among carriers, −0.15 [95% CI, −0.22 to −0.07]; *P* for interaction = .002) (Figure 2). In line with the results for cognition, we found that participants with prevalent dementia had higher values of skin autofluorescence (eTable 6 in the Supplement).

### Sensitivity Analyses

Similar results were obtained when using the alternative models for adjustment, including adjustment for age and sex only, and stratified by subcohort (Table 2; eTable 7 and eTable 8 in the Supplement). Exclusion of participants with diabetes or chronic kidney disease also did not change the results (eFigure 6 and eTable 9 in the Supplement), nor did adjustment for age squared. The results similarly did not change when restricting to dementia of the AD type (eTable 10 in the Supplement).

## Discussion

In this cohort study, we found that high EN-RAGE and low S-RAGE plasma levels were associated with higher dementia prevalence. High EN-RAGE plasma levels were also associated with an increased risk of incident dementia, but only for the short term. Tissue accumulation of AGEs, measured as skin autofluorescence, was associated with lower general cognitive function and *APOE* ε4 carrier status modified the association.

These results are in line with previous studies that reported cross-sectional associations of S-RAGE, EN-RAGE, and dementia, and with smaller sample size studies on skin, circulating, and urine AGEs and cognition.^6,41,42^ Our results suggest that the activation of RAGE by AGEs, EN-RAGE, or other ligands may be involved in the pathophysiological processes of dementia. Since the found associations were restricted to cross-sectional and short-term associations, reverse causation, meaning the exposure was causally related to the outcome, cannot be ruled out. For example, altered AGE and RAGE levels could be a consequence of lifestyle changes in the preclinical phase of dementia. However, skin AGEs, reflecting long-term AGE load, were associated with cognition in individuals without dementia, suggesting a role in cognitive decline. In line with this, we also found that participants with dementia had higher values of skin autofluorescence.

If AGEs and their receptor are associated with the risk of dementia, several putative mechanisms have been suggested, including disruption of the blood-brain barrier, facilitation of amyloid-β into the brain, vascular pathological processes, and activation of inflammatory pathways that subsequently leads to upregulation of RAGE expression.^4,11,12,43,44^ AGEs may also contribute to neurodegeneration by mechanisms independent of RAGE, such as modification and cross-linking of proteins, which may contribute to the toxic effects of amyloid-β,^45^ cellular damage and dysfunction, tissue stiffness, vascular pathological processes, and formation of aggregates.^2,3^

Because of the suggested involvement of RAGE in the pathological processes of dementia, and especially because of its suggested role in amyloid-β influx into the brain, inhibition of the amyloid-β–RAGE interaction was suggested to reduce the pathological processes of AD.^46^ Following this reasoning, a phase 3 clinical trial was conducted with patients with probable mild AD with an antagonist of RAGE but was ended earlier because futility criteria were met.^47^ However, whether inhibition of RAGE has a role in the prevention of dementia cannot be concluded from these data.

Regarding the long-term associations of EN-RAGE, the higher risk of dementia was diluted, especially in the high EN-RAGE group. Competing events, including mortality, may have concealed an adverse association of EN-RAGE with dementia because the high EN-RAGE group showed high mortality during follow-up and high EN-RAGE levels are associated with other diseases, such as coronary heart disease.^20^

Regarding S-RAGE, it is important to mention that 2 types of this molecule exist, both functioning as a decoy for RAGE ligands, that could not be distinguished by our measurement method.^48,49^ One of them, cleavage RAGE, is derived from cleavage of membrane-bound RAGE and is upregulated with RAGE activation. The second type is directly translated from alternative splicing of RAGE mRNA, therefore called endogenously secreted S-RAGE. Aside from a protective anti-inflammatory association, increased S-RAGE may therefore be a result of proinflammatory upregulation of RAGE. Future studies may consider distinguishing between the 2 types in their analyses or to focus on changes of levels over time to reduce this problem.

We observed a modification for the association between skin autofluorescence and cognitive function by *APOE* ε4 allele status, indicating that the AGE-RAGE system may participate in the pathological changes associated with *APOE*. *APOE* ε4 is associated with altered glucose metabolism in the central nervous system, and carriers of the *APOE* ε4 allele are more susceptible to stressors and injurious agents.^50^ It is less effective in prompting the proteolytic breakdown of amyloid-β aggregates and shows enhanced AGE-binding.^51,52,53,54^ In addition, differences in levels of AGE-RAGE markers by *APOE* ε4 carrier status were found in individuals who were not cognitively impaired.^55,56^ Summarized, *APOE* ε4 and the AGE-RAGE system may have a joint association with the pathophysiological processes of dementia.

Strengths of our study include the prospective design with a long follow-up, the extensive assessment of cognition using a cognitive test battery covering several domains, and near-complete dementia follow-up. Furthermore, skin AGEs were noninvasively measured in a large number of individuals. They are considered a proxy for AGEs in long-lived tissue, potentially including the brain, although no studies assessed their correlation.^42^

### Limitations

Limitations of this study include the absence of repeated measurements for cognition to assess cognitive decline, and of sufficient follow-up time for dementia after skin autofluorescence measurement. In addition, we may not have been able to detect modification of the associations between EN-RAGE and S-RAGE and dementia by *APOE* status, owing to small numbers within the strata. Furthermore, EN-RAGE and S-RAGE have a complex role both within and outside the AGE-RAGE system; therefore, they may not completely reflect the associations of the AGE-RAGE system with dementia. The measurement of circulating markers is also a momentary capture of the profile and may not adequately reflect the status later. Moreover, cardiometabolic risk factor management may have changed over time, which may have impacted the 3 cohort waves differently, potentially leading to bias. In this study, we were not able to examine the role of different diabetes medications, owing to limited sample sizes. Future studies are encouraged to investigate their role. Additionally, our results were restricted to an elderly population of European ancestry; future studies including participants of varying ethnicities and of younger age may extend the generalizability of the results.

## Conclusions

The results of this cohort study suggest that the AGE-RAGE system was associated with the pathophysiological processes of dementia. However, the association with the risk of dementia, if any, was restricted to the short term. Studies are warranted to investigate the potential for the AGE-RAGE system as a marker associated with future dementia.

## Notes

Supplement. **eFigure 1.** Flow Diagram for Study Population for Cross-Sectional and Longitudinal Analyses With EN-RAGE and S-RAGE **eFigure 2.** Flow Diagram for Study Population for Skin Autofluorescence and Cognition Outcomes **eFigure 3.** Timeline of Data Collection **eFigure 4.** Kaplan-Meier Survival Curves for Overall Survival by EN-RAGE Tertile **eFigure 5.** Kaplan-Meier Survival Curve for Overall Survival by S-RAGE Tertile **eFigure 6.** Association of Skin Autofluorescence and Cognition After Excluding Participants With Diabetes, Chronic Kidney Disease, or Both **eTable 1.** Summary of Missing Value in Covariates **eTable 2.** EN-RAGE and S-RAGE in Association With Dementia Prevalence and Incidence, Stratified by Sex and *APOE* ε4 Carrier Status **eTable 3.** EN-RAGE and S-RAGE and Dementia Incidence With Increasing Duration of Follow-Up **eTable 4.** Characteristics of the Skin Autofluorescence Cognition Study Population by Skin Autofluorescence Tertiles **eTable 5.** Scores of Cognitive Function Tests by Skin Autofluorescence Tertiles **eTable 6.** Skin Autofluorescence and Prevalent All-Cause Dementia in the Total Population **eTable 7.** Linear Regression Results for Association of Skin Autofluorescence With Cognition Adjusting for Different Sets of Covariates in the Total Study Population **eTable 8.** Association of Skin Autofluorescence and Cognition by Rotterdam Study Subcohorts (Model 2) **eTable 9.** EN-RAGE and S-RAGE in Association With Dementia Incidence After Excluding Participants With Diabetes, Chronic Kidney Disease, or Both **eTable 10.** EN-RAGE and S-RAGE in Association With Prevalent and Incident Alzheimer Disease
