# Supplementary material for: Assessment of Advanced Glycation End Products and Receptors and the Risk of Dementia
Source: JAMA Netw Open. 2021 Jan 8;4(1):e2033012. doi: 10.1001/jamanetworkopen.2020.33012 (PMC7794665; doi:10.1001/jamanetworkopen.2020.33012)
Supplement: Supplement. — eFigure 1. Flow Diagram for Study Population for Cross-Sectional and Longitudinal Analyses With EN-RAGE and S-RAGE eFigure 2. Flow Diagram for Study Population for Skin Autofluorescence and Cognition Outcomes eFigure 3. Timeline of Data Collection eFigure 4. Kaplan-Meier Survival Curves for Overall Survival by EN-RAGE Tertile eFigure 5. Kaplan-Meier Survival Curve for Overall Survival by S-RAGE Tertile eFigure 6. Association of Skin Autofluorescence and Cognition After Excluding Participants With Diabetes, Chronic Kidney Disease, or Both eTable 1. Summary of Missing Value in Covariates eTable 2. EN-RAGE and S-RAGE in Association With Dementia Prevalence and Incidence, Stratified by Sex and APOE ε4 Carrier Status eTable 3. EN-RAGE and S-RAGE and Dementia Incidence With Increasing Duration of Follow-Up eTable 4. Characteristics of the Skin Autofluorescence Cognition Study Population by Skin Autofluorescence Tertiles eTable 5. Scores of Cognitive Function Tests by Skin Autofluorescence Tertiles eTable 6. Skin Autofluorescence and Prevalent All-Cause Dementia in the Total Population eTable 7. Linear Regression Results for Association of Skin Autofluorescence With Cognition Adjusting for Different Sets of Covariates in the Total Study Population eTable 8. Association of Skin Autofluorescence and Cognition by Rotterdam Study Subcohorts (Model 2) eTable 9. EN-RAGE and S-RAGE in Association With Dementia Incidence After Excluding Participants With Diabetes, Chronic Kidney Disease, or Both eTable 10. EN-RAGE and S-RAGE in Association With Prevalent and Incident Alzheimer Disease [file jamanetwopen-e2033012-s001.pdf]

Supplemental Online Content

Chen J, Mooldijk SS, Licher S, et al. Assessment of advanced glycation end products and receptors and the risk of dementia. *JAMA Netw Open*. 2021;4(1):e2033012. doi:10.1001/jamanetworkopen.2020.33012

eFigure 1. Flow Diagram for Study Population for Cross-Sectional and Longitudinal Analyses With EN-RAGE and S-RAGE
eFigure 2. Flow Diagram for Study Population for Skin Autofluorescence and Cognition Outcomes
eFigure 3. Timeline of Data Collection
eFigure 4. Kaplan-Meier Survival Curves for Overall Survival by EN-RAGE Tertile
eFigure 5. Kaplan-Meier Survival Curve for Overall Survival by S-RAGE Tertile
eFigure 6. Association of Skin Autofluorescence and Cognition After Excluding Participants With Diabetes, Chronic Kidney Disease, or Both
eTable 1. Summary of Missing Value in Covariates
eTable 2. EN-RAGE and S-RAGE in Association With Dementia Prevalence and Incidence, Stratified by Sex and *APOE* ε4 Carrier Status
eTable 3. EN-RAGE and S-RAGE and Dementia Incidence With Increasing Duration of Follow-Up
eTable 4. Characteristics of the Skin Autofluorescence Cognition Study Population by Skin Autofluorescence Tertiles
eTable 5. Scores of Cognitive Function Tests by Skin Autofluorescence Tertiles
eTable 6. Skin Autofluorescence and Prevalent All-Cause Dementia in the Total Population
eTable 7. Linear Regression Results for Association of Skin Autofluorescence With Cognition Adjusting for Different Sets of Covariates in the Total Study Population
eTable 8. Association of Skin Autofluorescence and Cognition by Rotterdam Study Subcohorts (Model 2)
eTable 9. EN-RAGE and S-RAGE in Association With Dementia Incidence After Excluding Participants With Diabetes, Chronic Kidney Disease, or Both
eTable 10. EN-RAGE and S-RAGE in Association With Prevalent and Incident Alzheimer Disease

This supplemental material has been provided by the authors to give readers additional information about their work.

© 2021 Chen J et al. *JAMA Network Open.*

eFigure 1. Flow Diagram for Study Population for Cross-Sectional and Longitudinal Analyses With EN-RAGE and S-RAGE

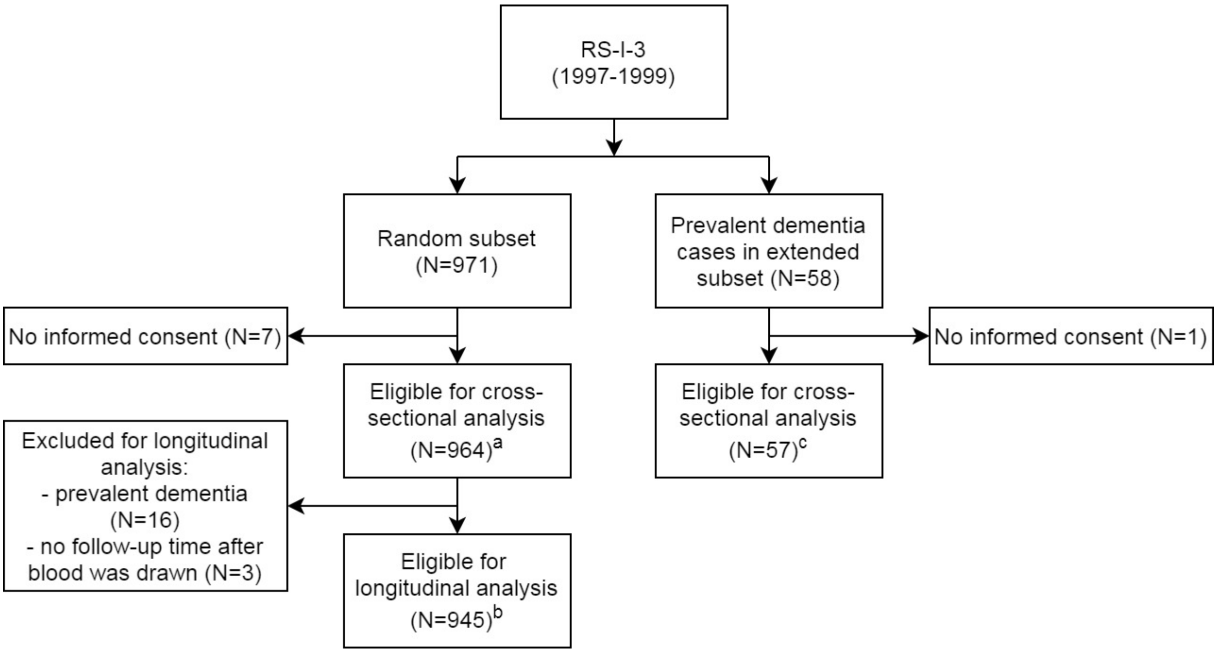

Flow diagram for participants of the EN-RAGE and S-RAGE subset.

[a] 1 participant had EN-RAGE missing, 2 had outlying EN-RAGE, leaving 961 eligible for prevalence analysis with EN-RAGE. There were no missing, or outlying values for S-RAGE.

[b] 1 participant had EN-RAGE missing, 2 had outlying EN-RAGE, leaving 942 eligible for incidence analysis with EN-RAGE. There were no missing, or outlying values for S-RAGE.

[c] 1 participant had EN-RAGE missing, 1 other participant had S-RAGE missing, leaving 56 eligible for prevalence analysis.

© 2021 Chen J et al. *JAMA Network Open.*

eFigure 2. Flow Diagram for Study Population for Skin Autofluorescence and Cognition Outcomes

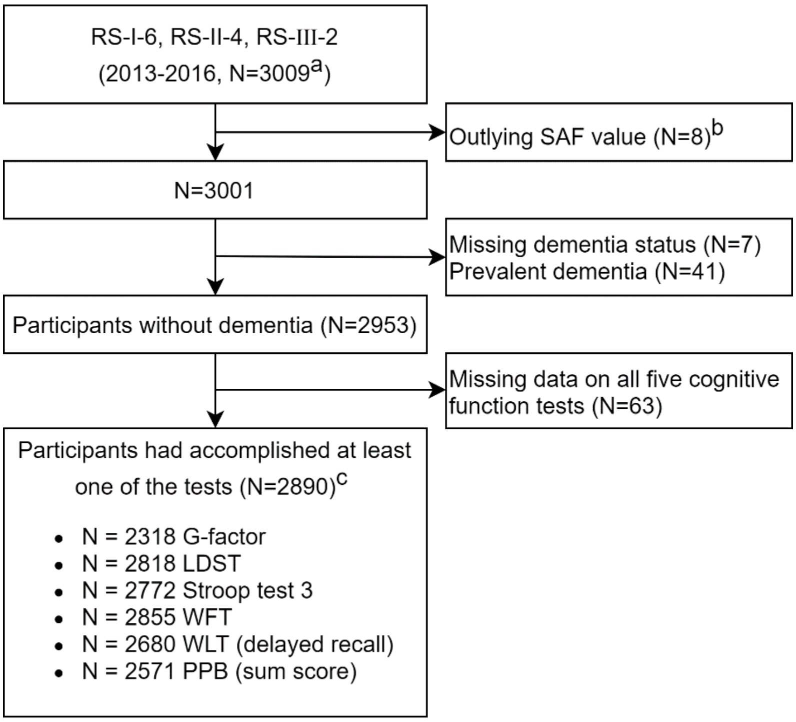

Flow diagram for participants of the SAF subset. SAF, skin auto-fluorescence; IC, informed consent; LDST, letter-digit substitution task; WFT, verbal fluency test; WLT (delayed recall), 15-word learning test (delayed recall); PPB, Purdue pegboard test.

[a] N=3029 had SAF measurement, of whom 20 with no informed consent were excluded.

[b] Values outside of the mean ± 4SD range were defined as outlying values.

[c] Participants with outlying cognitive test values were excluded for each analysis respectively (N=3 for G-factor, N=1 for LDST, N=1 for Stroop test 3, N=1 for PPB sum score).

eFigure 3 Timeline of data collection.

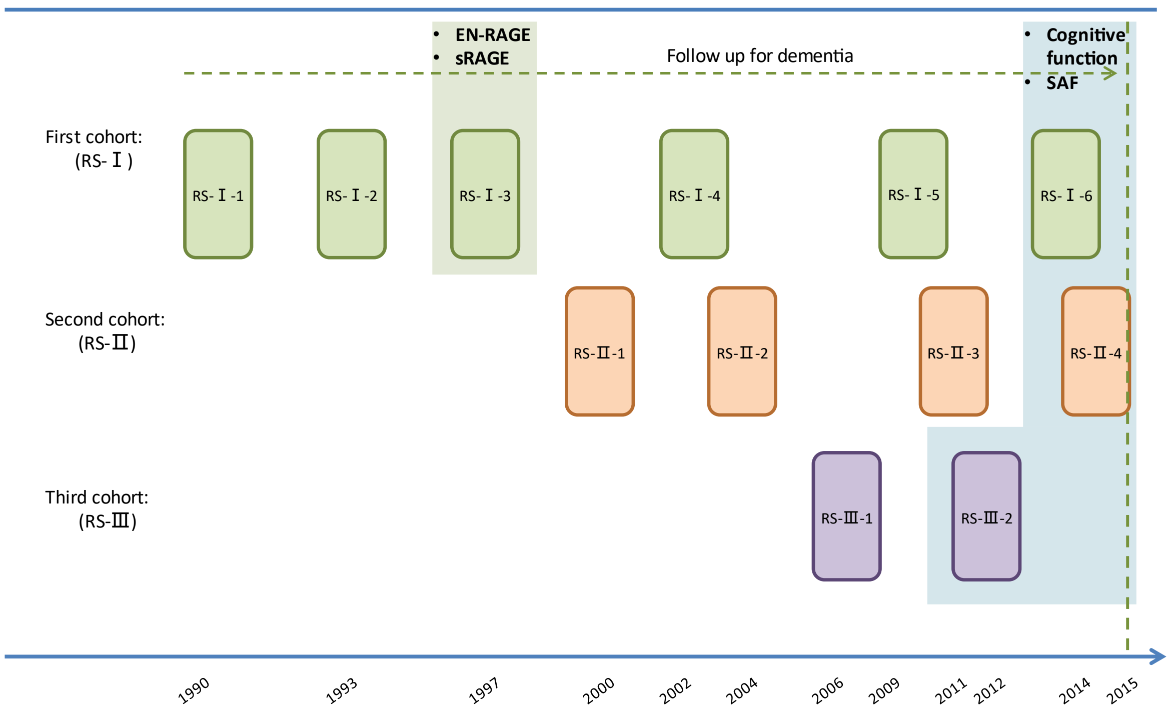

Abbreviations: EN-RAGE, Extracellular Newly identified RAGE binding protein; sRAGE, soluble form of the receptor for AGEs (RAGE); SAF, skin autofluorescence; RS, Rotterdam Study.
The number following each subcohort denotes the round of visit.

© 2021 Chen J et al. *JAMA Network Open.*

eFigure 4. Kaplan-Meier Survival Curves for Overall Survival by EN-RAGE Tertile

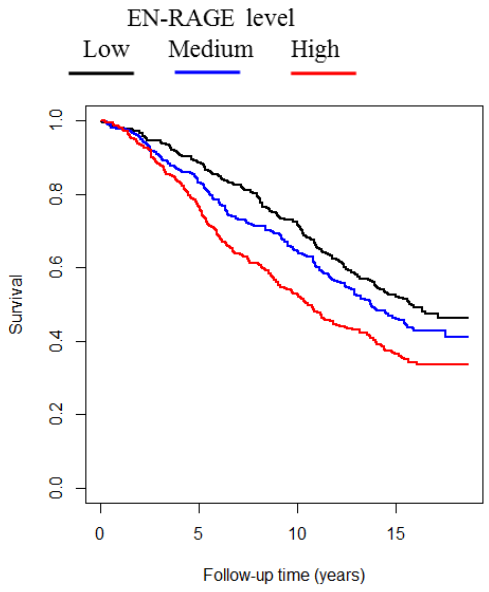

Follow-up time (years)	0	5	10	15
No. At risk				
Low	314	276	220	137
Medium	320	261	202	130
High	308	235	161	103

Unadjusted survival curves for overall survival by EN-RAGE tertile. Number of participants at risk at start of follow-up, after 5, 10 and 15 years, per tertile, are presented underneath the graph.

© 2021 Chen J et al. *JAMA Network Open.*

eFigure 5. Kaplan-Meier Survival Curve for Overall Survival by S-RAGE Tertile

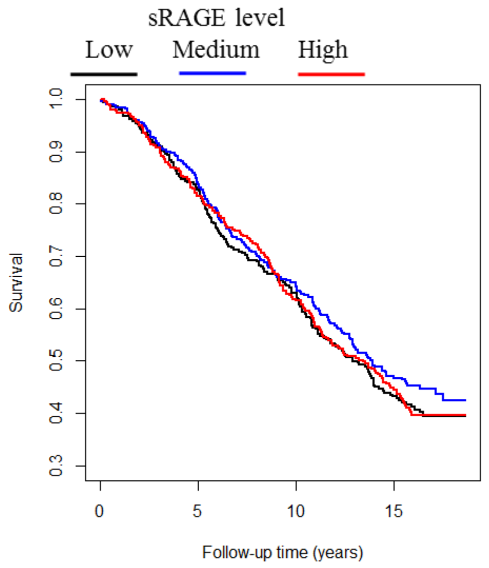

Follow-up time (years)	0	5	10	15
No. At risk				
Low	309	254	291	116
Medium	321	266	204	135
High	315	254	1689	120

Unadjusted survival curves for dementia free survival (left) and overall survival (right) by S-RAGE tertile. Number of participants at risk at start of follow-up, after 5, 10 and 15 years, per tertile, are presented underneath the graph.

© 2021 Chen J et al. *JAMA Network Open.*

eFigure 6 Association of SAF and cognition after excluding participants with diabetes, CKD or both.

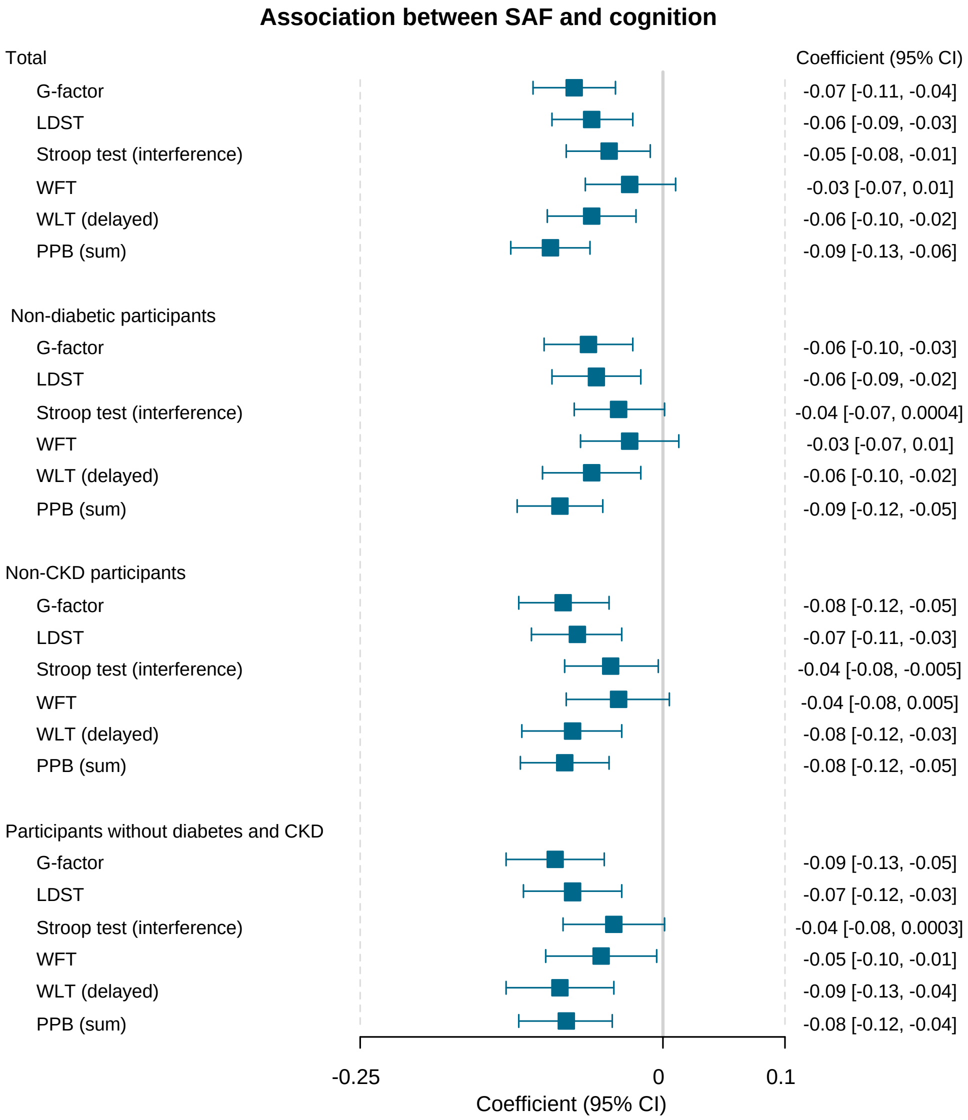

Abbreviations: CKD, chronic kidney disease; LDST, letter-digit substitution task; WFT, verbal fluency test; WLT, word learning test (delayed recall); PPB (sum), sum score Purdue pegboard tests including tests on left hand, right hand and both hands. Forest plot of the associations between z-score of SAF and G-factor and the performance of individual cognitive function tests in the total population and after excluding participants with diabetes, CKD or both. The original score of Stroop test (interference task) was inversely transformed. After transformation, higher score corresponds to better performance. Coefficients [95% CI]s are the difference in G-factor and z-scores of test parameters in association with one SD difference in SAF, obtained from the linear regression Model 2, adjusting for age, sex, diabetes, education, *APOE* ε4 carrier status, smoking status and eGFR. For the non-diabetic subgroups, diabetes status was not used in the model for adjustment.

© 2021 Chen J et al. *JAMA Network Open.*

eTable 1. Summary of Missing Value in Covariates

Variable	Percentage missing, %	
	**EN-RAGE and S-RAGE analyses**	**SAF analyses**
Diabetes status	9.6	0.8
Education	0.9	1.5
APOE ε4 carrier status	4.1	6.2
Smoking	2.1	1.2
eGFR	2.5	3.7
Systolic blood pressure	1.2	2.5
Diastolic blood pressure	1.2	2.5
HDL cholesterol	1.7	3.7
Total cholesterol	2.0	3.7
Triglycerides	2.5	3.7
Lipid lowering medication	10.0	0.8
Depressive symptoms	8.8	1.0

N = 1021 for EN-RAGE and S-RAGE analysis; N=2890 for SAF analysis. In total, 578 (14.8%) participants had missing value in at least one of the covariates, including 322 out of 2890 participants (11.1%) for the SAF analysis, and 256 out of 1021 (25%) participants for the EN-RAGE and S-RAGE analysis.

© 2021 Chen J et al. *JAMA Network Open.*

eTable 2. EN-RAGE and S-RAGE in Association With Dementia Prevalence and Incidence, Stratified by Sex and *APOE* ε4 Carrier Status

**A**	**Dementia prevalence**							
	**Women**		**Men**		***APOE* ε4 carriers**		***APOE* ε4 non-carriers**	
**EN-RAGE tertiles**	**n/N**	**OR[a] (95% CI)**	**n/N**	**OR[a] (95% CI)**	**n/N**	**OR[b] (95% CI)**	**n/N**	**OR[b] (95% CI)**
Low	6/197	1.00 Reference	3/127	1.00 Reference	4/67	1.00 Reference	5/250	1.00 Reference
Medium	11/180	1.72 (0.54-5.49)	5/157	0.75 (0.12-4.69)	11/114	1.45 (0.39-5.35)	4/211	0.88 (0.21-3.62)
High	27/185	2.82 (0.98-8.12)	20/171	4.38 (0.92-20.93)	25/117	2.68 (0.77-9.28)	18/216	3.40 (1.13-10.25)
Per SD Ln	44/562	1.41 (0.95-2.10)	28/455	2.30 (1.36-3.88)	40/298	1.50 (1.00-2.26)	27/677	1.79 (1.15-2.78)
	P = 0.20				P = 0.48			
	**Dementia incidence**							
	**Women**		**Men**		***APOE* ε4 carriers**		***APOE* ε4 non-carriers**	
**EN-RAGE tertiles**	**n/N**	**HR[a] (95% CI)**	**n/N**	**HR[a] (95% CI)**	**n/N**	**HR[b] (95% CI)**	**n/N**	**HR[b] (95% CI)**
Low	42/190	1.00 Reference	19/124	1.00 Reference	16/62	1.00 Reference	44/245	1.00 Reference
Medium	40/169	1.18 (0.74-1.86)	24/151	1.18 (0.62-2.25)	31/102	1.43 (0.77-2.66)	31/207	0.86 (0.53-1.37)
High	19/158	0.65 (0.37-1.15)	16/150	0.72 (0.35-1.48)	10/92	0.55 (0.24-1.26)	22/197	0.73 (0.43-1.24)
Per SD Ln	101/517	0.90 (0.73-1.11)	59/425	0.85 (0.62-1.15)	57/256	0.82 (0.61-1.11)	97/649	0.89 (0.71-1.10)
	P = 0.72				P = 0.39			

**B**	**Dementia prevalence**							
	**Women**		**Men**		***APOE* ε4 carriers**		***APOE* ε4 non-carriers**	
**S-RAGE tertiles**	**n/N**	**OR[a] (95% CI)**	**n/N**	**OR[a] (95% CI)**	**n/N**	**OR[b] (95% CI)**	**n/N**	**OR[b] (95% CI)**
Low	20/164	1.00 Reference	15/182	1.00 Reference	18/122	1.00 Reference	15/211	1.00 Reference
Medium	15/197	0.58 (0.24-1.42)	5/144	0.48 (0.13-1.76)	10/86	0.75 (0.27-2.06)	7/136	0.36 (0.13-1.01)
High	10/203	0.35 (0.13-0.97)	7/130	0.34 (0.09-1.25)	12/91	0.70 (0.27-1.86)	5/232	0.23 (0.07-0.78)
Per SD Ln	45/564	0.62 (0.41-0.93)	27/456	0.51 (0.30-0.86)	40/299	0.83 (0.53-1.30)	27/679	0.50 (0.33-0.76)
	P = 0.99				P = 0.06			

© 2021 Chen J et al. *JAMA Network Open.*

	Dementia incidence							
	**Women**		**Men**		***APOE* ε4 carriers**		***APOE* ε4 non-carriers**	
**S-RAGE tertiles**	**n/N**	**HR[a] (95% CI)**	**n/N**	**HR[a] (95% CI)**	**n/N**	**HR[b] (95% CI)**	**n/N**	**HR[b] (95% CI)**
Low	28/144	1.00 Reference	23/165	1.00 Reference	22/103	1.00 Reference	28/195	1.00 Reference
Medium	37/182	0.98 (0.59-1.63)	17/139	0.88 (0.45-1.72)	14/76	0.92 (0.45-1.87)	38/229	1.01 (0.61-1.65)
High	36/192	0.95 (0.57-1.58)	20/123	1.33 (0.70-2.53)	21/78	1.24 (0.65-2.37)	32/227	1.00 (0.59-1.68)
Per SD Ln	101/518	0.89 (0.72-1.11)	60/427	1.02 (0.77-1.33)	57/257	0.94 (0.69-1.28)	98/651	0.91 (0.74-1.13)
	P = 0.31				P = 0.61			

Associations of plasma EN-RAGE (A) and S-RAGE (B) levels with dementia prevalence and incidence. Odds ratio's (OR) and hazard ratio's (HR) are presented across tertile groups with the low-level group as reference; and by one standard deviation higher of natural logarithm of EN-RAGE and S-RAGE. All ORs were obtained from logistic regression and HRs from Cox regression, adjusting for age, sex, diabetes, education, *APOE* ε4 carrier status, smoking and eGFR (model 2). P-values for the corresponding interaction terms were calculated using the biomarkers continuously (per standard deviation of natural logarithm). The sample sizes for stratified analyses by *APOE* ε4 carrier status were smaller than those for sex stratified analyses because of missing value in *APOE* genotype. The slightly different sample sizes for EN-RAGE and S-RAGE were due to exclusion of outlying values.

[a]Sex was not used in the model for adjustment.

[b]*APOE* ε4 was not used in the model for adjustment.

© 2021 Chen J et al. *JAMA Network Open.*

eTable 3. EN-RAGE and S-RAGE and Dementia Incidence With Increasing Duration of Follow-Up

(A)

Follow-up duration	Events (low/medium/high)	HR (95% CI)	
		Medium EN-RAGE	High EN-RAGE
4	6/12/10	2.00 (0.73-5.48)	1.50 (0.52-4.29)
8	19/24/21	1.27 (0.68-2.39)	1.13 (0.59-2.15)
12	37/39/27	1.09 (0.68-1.73)	0.83 (0.50-1.40)
18.7	61/64/35	1.08 (0.76-1.55)	0.65 (0.42-1.00)

(B)

Follow-up duration	Events (low/medium/high)	HR (95% CI)	
		Medium S-RAGE	High S-RAGE
4	10/7/11	0.77 (0.28-2.09)	1.11 (0.45-2.75)
8	21/22/21	1.16 (0.62-2.17)	1.12 (0.60-2.12)
12	29/32/42	1.22 (0.72-2.07)	1.67 (1.02-2.75)
18.7	51/54/56	1.04 (0.70-1.55)	1.22 (0.82-1.81)

Associations of plasma EN-RAGE (A) and S-RAGE (B) levels with dementia incidence. HRs per cumulatively increasing duration of follow-up were obtained from Cox regression models by censoring all participants still at risk at 4, 8 and 12 years after baseline, and after total follow-up of 18.7 years. The group with the lowest plasma levels was used as a reference. All estimates were adjusted for covariates using Model 2, including age, sex, diabetes, education, *APOE* ε4 carrier status, smoking and eGFR.

© 2021 Chen J et al. *JAMA Network Open.*

eTable 4. Characteristics of the Skin Autofluorescence Cognition Study Population by Skin Autofluorescence Tertiles

	Total	Low SAF (1.10-2.13)	Medium SAF (2.17-2.50)	High SAF (2.53-4.40)
**N**	2890	959	897	1034
**Age**, year	72.51 ± 9.36	69.52 ± 9.46	72.37 ± 9.22	75.40 ± 8.46
**Sex** (Women)	1,640 (57)	650 (68)	498 (56)	492 (48)
**Caucasian descent** (n=2793)	2,675 (96)	873 (93)	840 (97)	962 (97)
**Education levels** (n=2846)				
Primary (n, %)	193 (7)	56 (6)	51 (6)	86 (8)
Lower (n, %)	1,120 (39)	370 (39)	342 (39)	408 (40)
Intermediate (n, %)	871 (31)	280 (30)	277 (31)	314 (31)
Higher (n, %)	662 (23)	241 (25)	215 (24)	206 (20)
**Income**, euro's/year (n=2596)				
<25000	187 (7)	49 (6)	58 (7)	80 (9)
25000-45000	767 (30)	197 (23)	244 (30)	326 (35)
45000-65000	756 (29)	233 (27)	243 (30)	280 (30)
>65000	886 (34)	374 (44)	262 (32)	250 (27)
***APOE* ε4** (n=2712)				
no alle (n, %)	1,977 (73)	638 (71)	621 (73)	718 (74)
1 alle (n, %)	672 (25)	237 (26)	210 (25)	225 (23)
2 alles (n, %)	63 (2)	20 (2)	22 (3)	21 (2)
**Alcohol use** (n=2858)	2,471 (86)	836 (88)	773 (87)	862 (84)
**Smoking status** (n=2854)				
Never (n, %)	916 (32)	371 (39)	280 (32)	265 (26)
Former (n, %)	1,565 (55)	501 (53)	482 (55)	582 (57)
Current (n, %)	373 (13)	79 (8)	122 (14)	172 (17)
**Body mass index**, kg/m^2 (n=2833)	27.54 (4.29)	27.18 (4.03)	27.53 (4.35)	27.89 (4.44)
**Systolic blood pressure,** mmHg (n=2817)	144.55 (21.52)	141.80 (20.76)	145.20 (21.97)	146.60 (21.58)
**Diastolic blood pressure**, mmHg (n=2817)	83.71 (10.79)	83.90 (10.76)	84.20 (10.85)	83.08 (10.74)
**Hypertension** (n=2853)	2,128 (75)	650 (68)	656 (74)	822 (81)
**Total cholesterol**, mg/dL (n=2782)	211 (42)	219 (44)	211 (41)	203 (42)
**High-density lipoprotein cholesterol**, mg/dL (n=2782)	58 (17)	61 (17)	58 (17)	56 (16)
*__Triglycerides__, mg/dL (n=2782)	113.37 (85.91, 153.23)	112.48 (86.80, 149.68)	110.71 (85.91, 149.90)	116.03 (86.80, 156.77)
**Lipid lowering medication** (n=2866)	877 (31)	246 (26)	261 (29)	370 (36)
*__eGFR__, mL/min/1.73 m^2 (n=2782)	74.92 (65.54, 84.41)	76.24 (67.56, 85.44)	75.33 (66.17, 84.61)	73.44 (62.86, 82.92)
**Chronic kidney disease** (n=2782)	413 (15)	99 (11)	118 (14)	196 (20)
**Diabetes** (n=2868)	393 (14)	82 (9)	114 (13)	197 (19)
**History of coronary heart diseases** (n=2878)	192 (7)	33 (3)	51 (6)	108 (11)
**Depressive symptom** (n=2862)	640 (22)	214 (22)	196 (22)	230 (22)
**SAF**, A.U.	2.40 ± 0.49	1.90 ± 0.19	2.32 ± 0.11	2.92 ± 0.35

Abbreviations: *APOE* = apolipoprotein E; eGFR = estimated glomerular filtration rate; SAF = skin autofluorescence.

© 2021 Chen J et al. *JAMA Network Open.*

SI conversion factor: To convert total and HDL cholesterol levels to mmol/L, multiply by 0.0259; To convert triglycerides levels to mmol/L, multiply by 0.0113.
Values are shown for non-imputed data. Values are counts (valid percentages), means (standard deviations) or *median (interquartile range) in case of a skewed distribution.

© 2021 Chen J et al. *JAMA Network Open.*

eTable 5. Scores of Cognitive Function Tests by Skin Autofluorescence Tertiles

	Number of included participants	Total (N=2890)	Low SAF (N=959)	Medium SAF (N=971)	High SAF (N=960)
**Stroop tests**					
***Stroop test 1**	2789	17.44 (15.63, 19.81)	16.84 (15.06, 18.91)	17.59 (15.66, 19.71)	18.17 (16.22, 20.73)
***Stroop test 2**	2788	23.59 (20.97, 26.81)	22.59 (20.32, 25.59)	23.66 (21.03, 26.57)	24.44 (21.94, 28.03)
***Stroop test 3**	2773	48.82 (40.75, 61.24)	44.97 (38.56, 57.19)	48.42 (40.58, 59.29)	53.07 (44.00, 67.25)
**Word fluency test**	2855	22.24 ± 5.80	23.07 ± 5.84	22.35 ± 5.70	21.30 ± 5.72
**Word learning test**					
**Word learning test (immediate)**	2692	7.72 ± 2.14	8.21 ± 2.15	7.72 ± 2.06	7.22 ± 2.09
**Word learning test (delayed)**	2680	7.10 ± 3.13	7.86 ± 3.14	7.14 ± 3.03	6.29 ± 3.02
***Word learning test (recognition)**	2668	14.00 (13.00, 15.00)	14.00 (13.00, 15.00)	14.00 (13.00, 15.00)	14.00 (12.00, 15.00)
**Purdue pegboard tests**					
**Purdue pegboard (both hands)**	2605	9.46 ± 2.02	10.09 ± 1.93	9.45 ± 1.92	8.80 ± 2.01
**Purdue pegboard (right hand)**	2664	12.29 ± 2.28	12.99 ± 2.14	12.36 ± 2.22	11.50 ± 2.24
**Purdue pegboard (left hand)**	2676	11.87 ± 2.22	12.48 ± 2.10	11.96 ± 2.16	11.15 ± 2.20
**Purdue pegboard (sum)**	2572	33.69 ± 5.77	35.61 ± 5.41	33.82 ± 5.55	31.57 ± 5.65
**Letter-digit substitution test**	2819	28.14 ± 7.01	29.78 ± 7.07	28.33 ± 6.77	26.29 ± 6.74
**G-factor**	2321	-0.19 ± 1.01	0.10 ± 0.95	-0.17 ± 0.97	-0.52 ± 1.01
**SAF, A.U.**	2890	2.40 ± 0.49	1.90 ± 0.19	2.34 ± 0.12	2.94 ± 0.35

Values are shown for non-imputed data. Values are counts (valid percentages), means ± standard deviations or *median (interquartile range) in case of a skewed distribution.

© 2021 Chen J et al. *JAMA Network Open.*

eTable 6. Skin Autofluorescence and Prevalent All-Cause Dementia in the Total Population

	**Without dementia (N=2953)**	**History of dementia (N=41)**		**OR (95% CI)**
Age, years	72.6 (9.3)	80.9 (6.2)	Model 1[a]	1.30 [0.96, 1.73]
Women, N (%)	1671 (57)	21 (51)	Model 2[b]	1.31 [0.96, 1.78]
SAF, A.U.	2.40 (0.49)	2.66 (0.49)	Model 3[c]	1.32 [0.97, 1.79]

Descriptive values are counts (valid percentages) or means (standard deviations). ORs (95% CIs) were obtained from N=2994 participants with known information on all-cause dementia (yes/ no) and represents the OR for dementia associated with one SD higher SAF.

[a] Model 1: the association was adjusted for age, sex, and diabetes.

[b] Model 2: the association was adjusted for age, sex, diabetes, education, *APOE* ε4 carrier status, smoking status and eGFR.

[c] Model 3: In addition to model 2, the association was further adjusted for potential mediators (systolic and diastolic blood pressure, cholesterol, HDL, triglycerides, lipid lowering medication, and depressive symptoms).

© 2021 Chen J et al. *JAMA Network Open.*

eTable 7. Linear Regression Results for Association of Skin Autofluorescence With Cognition Adjusting for Different Sets of Covariates in the Total Study Population

Outcome	N	Coefficient [95% CI]				
		**Crude**	**Age + sex[a]**	**Model 1[b]**	**Model 2[c]**	**Model 3[d]**
G-factor	2318	-0.29 [-0.33, -0.25]	-0.11 [-0.14, -0.07]	-0.10 [-0.13, -0.06]	-0.07 [-0.11, -0.04]	-0.07 [-0.10, -0.04]
LDST	2818	-0.24 [-0.27, -0.2]	-0.10 [-0.13, -0.06]	-0.08 [-0.12, -0.05]	-0.06 [-0.09, -0.03]	-0.06 [-0.09, -0.02]
Stroop test (interference)	2772	-0.22 [-0.25, -0.18]	-0.08 [-0.11, -0.04]	-0.07 [-0.10, -0.03]	-0.05 [-0.08, -0.01]	-0.04 [-0.08, -0.01]
WFT	2855	-0.14 [-0.17, -0.1]	-0.05 [-0.09, -0.02]	-0.05 [-0.08, -0.01]	-0.03 [-0.07, 0.01]	-0.03 [-0.07, 0.01]
WLT (delayed)	2680	-0.22 [-0.26, -0.18]	-0.08 [-0.12, -0.05]	-0.07 [-0.11, -0.04]	-0.06 [-0.10, -0.02]	-0.06 [-0.09, -0.02]
PPB (sum)	2571	-0.32 [-0.35, -0.28]	-0.12 [-0.15, -0.09]	-0.11 [-0.14, -0.08]	-0.09 [-0.13, -0.06]	-0.09 [-0.12, -0.05]

Abbreviations: LDST, letter-digit substitution task; WFT, verbal fluency test; WLT, word learning test; PPB (sum), sum score Purdue pegboard tests including tests on left hand, right hand and both hands.

Coefficients and respective 95% CIs were obtained from linear regression models with the z-score of SAF as independent variable, representing adjusted difference in G-factor and z-scores of test parameters associated with one SD difference in SAF.

[a] Age + sex: the association was adjusted for age and sex.
[b] Model 1: the association was adjusted for age, sex and diabetes.
[c] Model 2: the association was adjusted for age, sex, diabetes, education, *APOE* ε4 carrier status, smoking status and eGFR.
[d] Model 3: In addition to model 2, the association was further adjusted for systolic and diastolic blood pressure, cholesterol, HDL, triglycerides, lipid lowering medication, and depressive symptoms.

© 2021 Chen J et al. *JAMA Network Open.*

eTable 8. Association of Skin Autofluorescence and Cognition by Rotterdam Study Subcohorts (Model 2)

**Cognitive function test**	**Coefficient [95% CI]**		
	**RS I** (N=703)	**RS II** (N=1036)	**RS III** (N=1151)
G-factor	-0.12 [-0.19, -0.05]	-0.06 [-0.12, -0.01]	-0.12 [-0.17, -0.07]
LDST	-0.08 [-0.14, -0.01]	-0.06 [-0.12, -0.01]	-0.12 [-0.18, -0.06]
Stroop test (interference)	-0.08 [-0.14, -0.02]	-0.04 [-0.1, 0.02]	-0.1 [-0.15, -0.04]
WFT	-0.08 [-0.15, -0.01]	-0.01 [-0.07, 0.05]	-0.05 [-0.12, 0.01]
WLT (delayed)	-0.09 [-0.16, -0.03]	-0.05 [-0.11, 0.01]	-0.09 [-0.15, -0.03]
PPB (sum)	-0.08 [-0.14, -0.02]	-0.12 [-0.18, -0.07]	-0.13 [-0.19, -0.08]

Abbreviations: CI, confidence interval; RS, Rotterdam Study; LDST, letter-digit substitution task; WFT, verbal fluency test; WLT (delayed), word learning test (delayed recall); PPB (sum), sum score Purdue pegboard tests including tests on left hand, right hand and both hands.

N, the number of participants who had accomplished at least one of the tests. The actual sample size of analysis for each cognitive function test may be less than the total population of the subcohort as not every participant underwent the full test battery.

Coefficients [95% CI]s are the difference in G-factor and z-scores of test parameters in association with one SD difference in SAF, obtained from the linear regression model 2, adjusting for age, sex, diabetes, education, APOE ε4 carrier status, smoking status, and eGFR.

© 2021 Chen J et al. *JAMA Network Open.*

eTable 9. EN-RAGE and S-RAGE in Association With Dementia Incidence After Excluding Participants With Diabetes, Chronic Kidney Disease, or Both

(A)

	Diabetics excluded			CKD excluded			Diabetics and CKD excluded	
**EN-RAGE tertiles**	**n/N**	**HR* (95% CI)**		**n/N**	**HR (95% CI)**		**n/N**	**HR* (95% CI)**
Low	50/265	1.00 Reference		48/262	1.00 Reference		38/223	1.00 Reference
Medium	47/260	1.00 (0.66-1.50)		57/271	1.21 (0.81-1.79)		43/219	1.22 (0.78-1.90)
High	24/221	0.69 (0.42-1.14)		26/229	0.64 (0.39-1.06)		18/165	0.75 (0.42-1.34)
Per SD Ln	121/746	0.89 (0.74-1.09)		131/762	0.88 (0.73-1.07)		99/607	0.95 (0.77-1.17)

(B)

	Diabetics excluded			CKD excluded			Diabetics and CKD excluded	
**S-RAGE tertiles**	**n/N**	**HR* (95% CI)**		**n/N**	**HR (95% CI)**		**n/N**	**HR* (95% CI)**
Low	39/234	1.00 Reference		43/264	1.00 Reference		38/223	1.00 Reference
Medium	40/253	0.99 (0.62-1.57)		46/264	1.09 (0.71-1.69)		43/219	0.97 (0.59-1.60)
High	43/262	1.09 (0.69-1.72)		43/237	1.32 (0.85-2.06)		18/165	1.08 (0.65-1.79)
Per SD Ln	122/749	0.93 (0.76-1.14)		132/765	0.98 (0.81-1.18)		99/607	0.91 (0.73-1.15)

Associations of plasma EN-RAGE (A) and S-RAGE (B) levels with dementia incidence. HRs are presented across tertile groups with the low-level group as reference; and by one standard deviation higher of natural logarithm of EN-RAGE and S-RAGE. All HRs were obtained from Cox regression for dementia incidence, adjusting for age, sex, diabetes, education, *APOE* ε4 carrier status, smoking and eGFR (model 2).

*Diabetes status was not used in the model for adjustment.

© 2021 Chen J et al. *JAMA Network Open.*

eTable 10. EN-RAGE and S-RAGE in Association With Prevalent and Incident Alzheimer Disease

(A)

EN-RAGE tertiles	Cross-sectional			Longitudinal	
	n/N	OR (95% CI)		n/N	HR (95% CI)
Low	6/324	1.00 Reference		48/314	1.00 Reference
Medium	14/337	2.02 (0.68-6.00)		54/320	1.18 (0.79-1.76)
High	36/356	3.61 (1.33-9.78)		27/308	0.67 (0.41-1.09)
Per SD Ln	56/1017	1.54 (1.10-2.16)		129/942	0.90 (0.75-1.08)

(B)

S-RAGE tertiles	Cross-sectional			Longitudinal	
	n/N	OR (95% CI)		n/N	HR (95% CI)
Low	25/346	1.00 Reference		41/309	1.00 Reference
Medium	18/341	0.80 (0.37-1.75)		43/321	1.01 (0.65-1.59)
High	14/333	0.50 (0.21-1.15)		46/315	1.22 (0.78-1.89)
Per SD Ln	57/1020	0.72 (0.52-1.00)		130/945	0.99 (0.82-1.20)

Associations of plasma EN-RAGE (A) and S-RAGE (B) levels with Alzheimer's disease (AD). Results were obtained from logistic models for prevalent AD and from Cox regression for incident AD. The group with the lowest plasma levels was used as a reference. All ORs and HRs were adjusted using model 2, including age, sex, diabetes, education, *APOE* ε4 carrier status, smoking and eGFR.

© 2021 Chen J et al. *JAMA Network Open.*
